# Water Vapor Permeability through Porous Polymeric Membranes with Various Hydrophilicity as Synthetic and Natural Barriers

**DOI:** 10.3390/polym12020282

**Published:** 2020-02-01

**Authors:** Chalykh Anatoly, Zolotarev Pavel, Chalykh Tatiana, Rubtsov Alexei, Zolotova Svetlana

**Affiliations:** 1 Frumkin Institute of Physical Chemistry and Electrochemistry of Russian Academy of Sciences, Leninsky Prospect, 31, bldg. 4, Moscow 119071, Russia; usecretar@phyche.ac.ru; 2 Department of Commodity Science and Expertise, Plekhanov Russian University of Economics, Stremyanny per. 36, Moscow 117997, Russia; TCHalykh.TI@rea.ru (C.T.); ealexru@gmail.com (R.A.); goldoni@yandex.ru (Z.S.)

**Keywords:** permeability, diffusion, sorption, porous membranes, hydrophilic and hydrophobic polymers

## Abstract

The article is devoted to the analysis of sorption kinetics, permeability, and diffusion of water vapor in porous polymeric membranes of different hydrophilicities and through-porosities. The water transport measurement with a constant gradient of partial pressure allows the authors to obtain reliable characteristics for porous membranes, films, artificial leathers, and fabrics of various chemical natures (synthetic and bio-based) and phase structures. All the kinetic permeability curves were determined and effective diffusion coefficients, as well as their apparent activation energies, were calculated at the stationary and non-stationary stages of the mass transfer. The relationship between the sorption–diffusion characteristics of the polymer barriers and their vapor permeability is traced. Within the framework of a Zolotarev–Dubinin dual dispersive model, an analytical equation is obtained that relates permeability to diffusion coefficients of water vapor in the pore volume, polymer skeleton material using such characteristics as porosity and the solubility coefficient. It is proposed to use this equation to predict the sorption properties for barrier and porous materials of complex architecture specifically in food packaging.

## 1. Introduction

Polymeric materials of different chemical natures and phase structures have such diverse functional purposes that the lifetime of products based on them varies drastically. It is natural that the products for long-term use, which are subject to high loads during operation, should be durable and resistant to environmental factors. Food packaging materials and containers, on the contrary, should be used no longer than the expiration period of the foodstuff. Otherwise, such materials, being discarded as solid domestic waste, pollute the environment. It is known that polymeric materials with short shelf life should be decomposable [1]. The best conditions for biodegradation of polymers are created in humid conditions, when moisture is immobilized in hydrophilic polymers, creating conditions for the development of microflora [2]. This process is significantly accelerated in porous polymeric membranes. Therefore, information on the interaction of moisture with polymers is of fundamental importance for solving the urgent problems of material science such as the choice of polymers for specific aims and the prediction of the behaviour of materials in the processes of saturation by water and biodegradation [3,4]. This article is devoted to the analysis of the process of sorption and diffusion of water vapor in porous polymers of different hydrophilicities.

From the standpoint of percolation theory, traditional analysis of the permeability of gas-filled polymers, and heterogeneous polymeric hybrid systems in general, can be divided into two states: Before the percolation transition, when the polymeric phase forms a continuous dispersion medium, and gas phase forms closed inclusions, and after the percolation transition, when the gas phase forms channels through the other phase. While the process phenomenology is sufficiently developed for the first state, much remains unclear for the second state [5,6]. First of all, what contribution to the transfer process is made by the matrix, its sorption and diffusion characteristics, tortuosity of transport channels, adsorption at the interface. The question of the methods of investigation of vapor permeability of these materials remains also open. Currently, the cup method has become the most widespread for the experimental determination of water vapor permeability through porous polymeric membranes. In this method, the value of flow *I* in the stationary area of the kinetic permeability curve is the main characteristic of the membrane, and it is usually identified with the permeability coefficient. This method is used successfully for standard permeability measurements of barrier materials.

On the other hand, the non-stationary part of the curve, within which the concentration distribution of the diffusant over the membrane cross-section is established, remains out of the attention of the researchers, although it is this very part that bears the greatest information on the features of water vapor diffusion through porous and monolithic polymeric materials [1,7]. Thus, the non-stationary area can be used to calculate the diffusion coefficient (*D*), to estimate its concentration and temporal dependencies, and to determine the solubility coefficients and Henry’s constants (*s*). Unfortunately, the cup method in its modern version [8] does not allow the obtainment of information on the non-stationary part of the permeability curve since a gradient of the total pressure of the external environment is present in chromatographic and mass spectrometric cells along with the gradient of the partial pressure. Therefore, its use in porous membranes to study the mass transfer in isobaric–isothermal conditions in detail is problematic.

The purpose of this work was to study the kinetics of water vapor permeability through porous polymeric membranes of different hydrophilicity by continuous registration of the vapor diffusate amount, combined with the theoretical analysis of the mass transfer process in such systems.

## 2. Experimental

Monolithic and porous polymeric membranes with thicknesses of 150 μm and 500 μm made of collagen (C), polyesterurethane urea (PEU), polyamide 6.6 (PA), polyvinyl chloride (PVC) were used as the study objects. PEU membranes were obtained from the 4,4-diphenylmethane diisocyanate, polyetherdiols, and hydrazinehydrates dimethylformamide solution. Oligooxypropylenediol was used in the synthesis of PEU-1, statistical copolymer of propylene and ethylene oxides in the ratio of 1:1 for the synthesis PEU-2, and an ester of adipic acid and oxypropylenediol were used for PEU-3.

PEU of Sanpren brand (Japan) (M_W_ = 32.9 kDa, M_n_ = 22.1 kDa) was obtained from a solution in dimethylformamide of 4,4-diphenylmetandiisocyanate, polyesterdiols and hydrazine hydrates. Oligooxypropyleneddiol was used in the synthesis of PEU-1, and a statistical copolymer of propylene and ethylene oxides in the ratio of 1:1 was used for PEU-2, and for PEU-3—a complex ester of adipic acid and oxypropylenediol.

PVC monolithic films of C-70 brand (Nizhny Novgorod, Russia) (M_W_ = 107.2 kDa) were obtained by the casting of 5% wt% of polymer solution onto a glass substrate with subsequent drying of films to a constant weight.

Collagen membranes (M_W_ = 360 kDa) were obtained from acetic acid solution of the soluble part of animal dermis (Moscow, Russia). Fixed collagen film was washed from acetic acid residues in distilled water.

Porous PEU was obtained from 10 wt% solution in dimethylformamide by the condensation-induced structure formation method [9]. Water was used as a precipitator. Porous PVC films were obtained by its deposition from 5 wt% solutions in cyclohexanone via precipitation by ethanol, and PA films obtained from its solutions in ethanol via precipitation by water. The porous analogue of collagen was a sample of natural leather (raw and unpainted calfskin). The porosity of the samples and their specific surface were determined by measuring the density and sorption of the inert solvent, hexane. Characteristics of the studied samples are given in Table 1.

**Figure 1:**
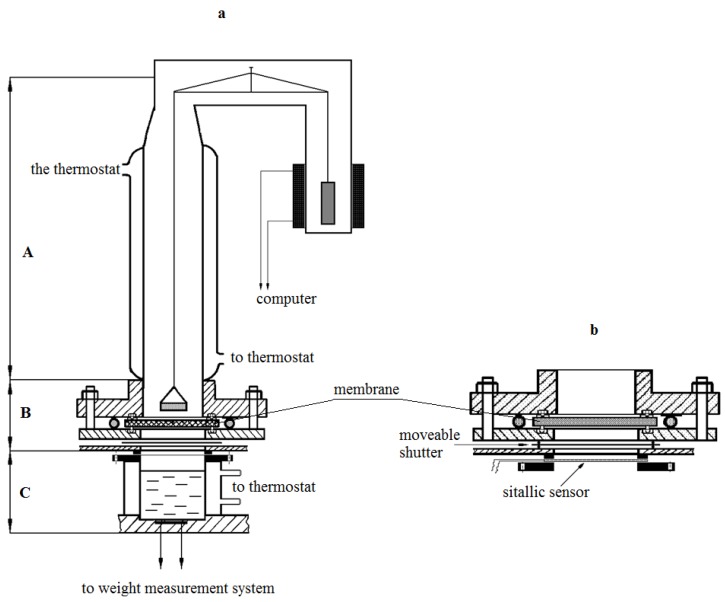
Experimental setup (**a**) and diffusion cell (**b**) for the study of the vapor permeability kinetics of polymeric membranes. Sitallic sensor is designed to measure the local humidity near the inner surface of the membrane. More detailed description of the setup is provided in the text.

Isotherms of water vapor sorption were obtained on McBain’s vacuum scales. The kinetics of water vapor permeability were studied using an experimental setup, the scheme of which is shown in Figure 1. The unit consists of a vapor source block (C), diffusion cell (B), which houses the membrane under study between the metal flanges, glass sorption column with quartz spiral, and a cup with water vapor absorber. In contrast to the previously created setups [8,9], the source block contains several glass thermostable cups mounted on a movable plastic disc.

Each source was filled with an aqueous salt solution before the experiment, in this case, CaCl_2_. The composition of the solutions was chosen in such a way that it would be possible to change the gradient of partial pressure of steam from (p/p_0_)_1_ to (p/p_0_)_2_ by changing the sources, moving along the sorption isotherm. Here, (p/p_0_)_1_ and (p/p_0_)_2_ are relative humidity values above the source and the sink, respectively.

The disc is equipped with a special lifting device, with the help of which a tight connection was made between the cup and the metal flange of the cell. The distance from the membrane surface to the source is 2 cm, from the cup with absorber to the membrane surface is 2.5 cm. All units of the setup have independent temperature control, which allows us to carry out measurements both in isothermal and non-isothermal modes in a wide range of partial steam pressures.

The measurement method was as follows. The membrane sample after long conditioning was removed from the desiccator, in which the same absorber was used as a hydrostat (for example, K_2_CO_3_ provides a constant humidity of ≈44%), and then was installed between the flanges of the diffusion cell. The absorber was placed in the sorption column. The establishment of sorption equilibrium in the membrane-absorber system at the chosen temperature of the experiment was observed during the next 10–20 min. Then the block of sources (lower block) was brought into contact with the lower flange of the diffusion cell. From this moment on, the kinetics of the absorber weight change were measured. These measurements continued until a constant absorber weight change rate was reached, which corresponds to the establishment of a stationary state of the transfer process at the selected partial pressure of water vapor.

The design of the setup allowed to change the water vapor partial pressure difference measurements range via selection of the source (Figure 1b). The relative error in determining the kinetics of water vapor permeability at the stationary stage was 5%, and 8% at the non-stationary stage. Measurements were carried out in isothermal conditions of the process at temperatures of 298–230 K at various moisture differences between the source and absorber.

## 3. Results and Discussion

**Figure 2:**
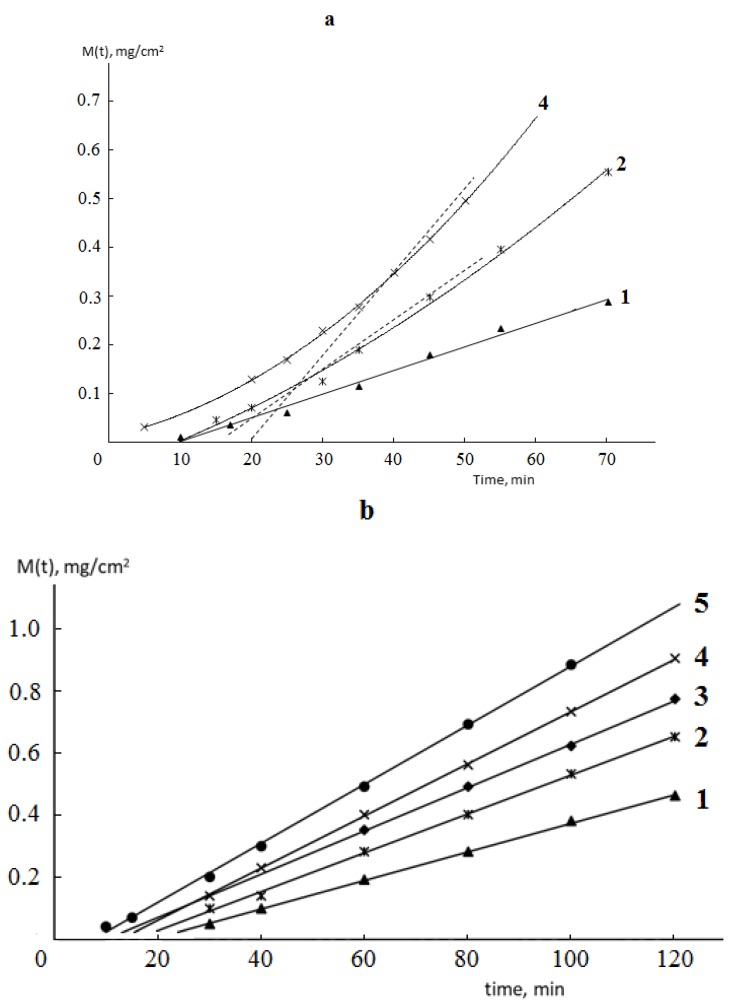
Kinetic curves of vapor permeability of monolithic (**a**) and porous (**b**) polymeric membranes obtained from polyvinyl chloride (PVC) (**1**), polyesterurethane urea (PEU)-1 (**2**), PEU-2 (**3**), PEU-3 (**4**), and collagen (**5**) at 298 K.

**Figure 3:**
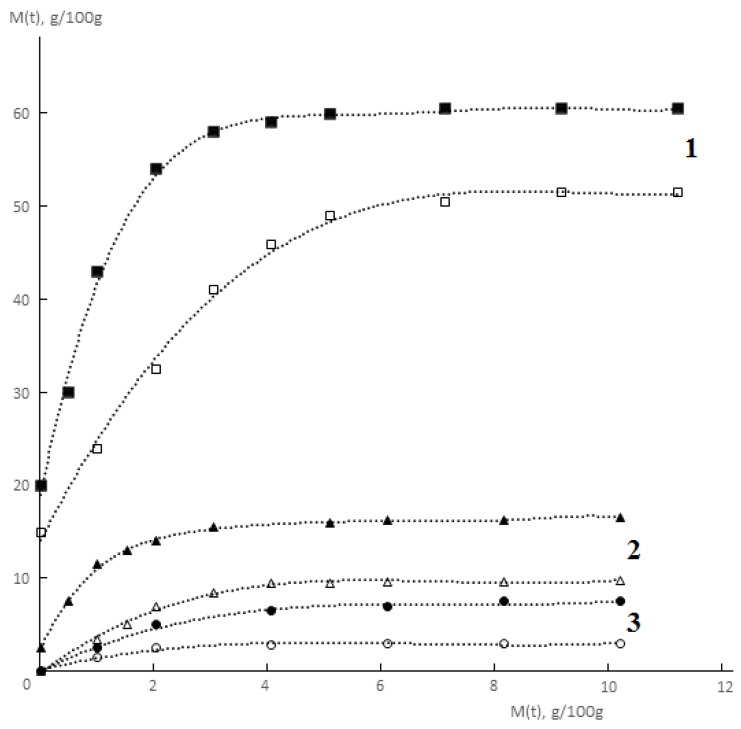
Kinetic curves of water vapor sorption by monolithic (**solid lines**) and porous (**dotted lines**) membranes made of collagen (**1**), PEU-3 (**2**), PEU-1 (**3**) at 298 K. Interval of relative water vapor pressure difference is 0.44–0.90.

Typical kinetic curves of permeability and sorption of water vapor by monolithic and porous membranes of different hydrophilicity are shown in Figure 2 and Figure 3. It is seen that for both monolithic and porous membranes with through-porosity, two areas corresponding to the non-stationary and stationary stages of the process can be distinguished on the kinetic curves of permeability.

From the formal point of view, regardless of the phase state of the membrane and its porosity, the length of the non-stationary stage can be characterized by the time delay (Θ), which is numerically equal to the segment cut off on the time axis by the extrapolated linear part of the permeability curve (Figure 2).

For porous membranes, Θ is always smaller than that for monolithic membranes and is significantly larger than that for the “source/air layer/absorber” system. For the latter, Θ is equal to 10–12 s, which, according to Barrer, gives the value of diffusion coefficient *D* = 0.2 cm^2^/s, which coincides under normal conditions with the diffusion coefficient of water vapor in the air [10].

**Figure 4:**
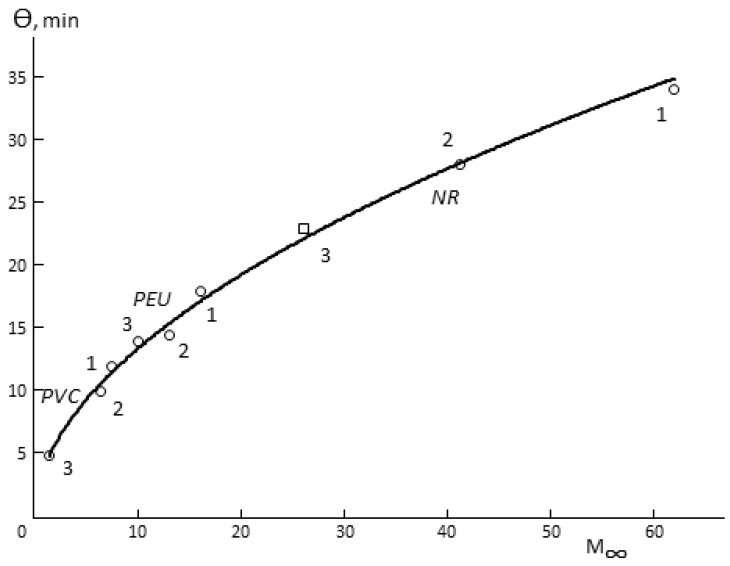
Dependence of the delay time (Θ) on the sorption capacity of the porous polymeric membranes (M∞) at 298 K, relative humidity of 30% at various values of relative humidity differences: (**1**) 44–90 p/p_0_; (**2**) 44–80 p/p_0_; (**3**) 44–60 p/p_0_. Polymer materials are denoted on the graph.

The following three facts are interesting. First, Θ for porous membranes is a function of hydrophilicity (Figure 4). In spite of the fact that the total porosity of the samples changes in quite a wide range (Table 1), it can be argued that Θ increases together with increasing of the sorption capacity of the polymer, from which the membrane is made. Changes in the humidity difference upon the measurement of vapor permeability shift the figurative point of the sample in one or another direction on the Θ*-M* (*∞*) curve. This effect is not observed for monolithic membranes.

Secondly, there is a proportionality between Θ and the time of sorption equilibrium establishment in the membrane–water vapor system.

Thirdly, the ratios between the diffusion coefficients and temperature coefficients for porous and monolithic membranes, calculated from the stationary area of the permeability curve and the sorption kinetics, are unusual. Diffusion coefficients for monolithic membranes can be calculated by the following traditional equations [9,10,11]:

D_Θ_ = l^2^/6 Θ (1)

D_s_ = (π/16) l^2^ (*d*γ/*d*t^1/2^)^2^ (2)

D_p_ = I/σ ^−1^ (3)

where *l* is the membrane thickness and γ is the relative degree of the membrane filling by diffusant.

While, the thus obtained diffusion coefficient values, as well as the apparent sorption and permeability activation energies E_Θ_, E_s_, E_p_, (Table 1), are quite close to each other, the situation is completely different for the case of porous membranes.

The effective diffusion coefficients for porous membranes D_Θ_′ D_s_′ D_p_′, calculated using the same Equations (1)–(3), differ significantly among themselves (Table 1). For all humidity differences, as a rule, (D_Θ_′ = D_s_′) << D_p_, and for absolute values D_Θ_′ >> D_Θ_. For hydrophilic membranes (collagen, PEU-2, and PEU-3), the difference between D_Θ_′ and D_p_′ is greater the greater the humidity difference. This effect is determined by the decrease of D_Θ_′ to a greater degree than by the increase of D_p_′ and *I*. Thus, upon transition from humidity difference of 44–60% to 44–90%, D_Θ_′ decreases by 2.5 times, and D_p_′ and *I* increase only by 30–35% due to the plasticization of polymer.

The temperature dependences of D_Θ_′ and D_s_′ for all membranes studied are described satisfactorily by the Arrhenius equation. At the same time, E_Θ_′ and E_s_′ are close to each other and, especially importantly, coincide with E_Θ_ and E_s_ for monolithic membranes. This means that the main contribution to the mass transfer at the non-stationary stage is made by the process of sorption saturation of the porous membrane frame by the diffusant.

However, the temperature dependence of permeability of porous membranes in the general case does not obey the Arrhenius equation. For PVC and PEU-1 membranes at all (p/p_o_), and for collagen and PEU-3 membranes at (p/p_o_)_1_ < 60%, the temperature dependences of *I* are described by the power function

I ≅ k T^n^ (4)

where *n* is an empirical constant, the value of which varies from 1.5 to 1.7, which indicates a significant contribution of free diffusion of water vapor through the pore space of the membranes to the stationary permeability. In the area of high humidity (more than 80%), the temperature dependence of water vapor permeability for collagen, PEU-2, and PEU-3 is described by a set of two functions

I ≅ k_1_ T^n^ + k_2_ exp (−E_Θ_/RT) (5)

where k_1_ and k_2_ are empirical constants.

The experimental data obtained allow us to formulate the following ideas about the mechanism of vapor permeability of porous polymeric membranes. The process of moisture transfer in such membranes is a superposition of two flows: Phase transfer over the porous space of the membrane and diffusion transfer over the volume of the polymeric matrix (called activation diffusion according [10] or solid-state diffusion according to [9,11]). At t < Θ, water vapor penetrates into the membrane through the system of through capillaries. Simultaneously with the diffusion through the free space of pores, vapor is adsorbed on the pore walls and absorbed–dissolved in the wall material. This process occurs before local sorption equilibrium is established in different parts of the membrane, and its rate is determined by the coefficient of water vapor diffusion into the polymer.

At t > Θ, some humidity gradient is set over the whole thickness of the sample. This moment corresponds to the beginning of the stationary stage of the process, in which both the pore space and the membrane material are involved simultaneously. Thus, until a constant moisture gradient is established over the cross-section of the membrane, most of the flow is absorbed by the material and does not participate in the direct transfer of water vapor to the outer surface of the sample. Once a constant gradient has been established, the flows through the porous space and polymer material become parallel, and the total flow is determined by their sum [10,11].

The theory of the mass transfer process in porous materials is described in detail in the following work’s papers [12,13].

The averaged equations of mass transfer in the above described systems with a developed system of transport pores are as follows:

(6) $$m\frac{\partial c}{\partial t}=mD_{i}\frac{\partial^{2}c}{\partial x^{2}}-\kappa \left(\gamma c-a\right)$$

(7) $$\left(1-m\right)\frac{\partial c}{\partial t}=\left(1-m\right)D_{a}\frac{\partial^{2}a}{\partial x^{2}}+\kappa \left(\gamma c-a\right)$$

(8) $$c\left(0,t\right)=c_{0},\;\;a\left(0,t\right)=\gamma c_{0},\;\;a\left(l,t\right)=c\left(l,t\right)=0$$

(9) a(x,0)=c(x,0)=0,  0≤x≤l

where m is the porosity of the membrane, *l* is its thickness, *c* and *a* are the local concentrations of the diffusant in the pores and the polymeric phase, D_i_ and D_a_ are the diffusion coefficients in the pores and the polymeric phase, respectively, k is the averaged constant of the mass transfer rate between the diffusant in the pores and the polymeric phase.

By introduction of dimensionless variables and parameters

(10) $$\xi =\frac{x}{l}\;\;\;\;\varepsilon =\frac{\tau_{i}}{\tau_{a}}\;\;\beta =\kappa \tau_{i}/\left(1-m\right)\;\;\alpha =\kappa \sigma \tau_{i}/m\;\;\;\tau_{i}=\frac{l^{2}}{D_{i}}\;\;\;\tau_{a}=\frac{l^{2}}{D_{a}}\;\;\tau =\frac{t}{\tau_{i}}$$

we obtain the following equation for the amount of substance $M\left(t=\right)\int_{0}^{t}q\left(t\right)dt$, desorbed from the membrane at the moment t (i.e., M(t) absorbed by the sorbent) from the surface

(11) $$M\left(t\right)=\frac{mD_{i}+\left(1-m\right)\sigma D_{a}}{l}c_{0}\times \times \left\{t-t_{1}-2\tau_{i}\overset{\infty }{\underset{n=1}{\sum }}\frac{{\left(-1\right)}^{n}}{\left(\lambda_{n}^{-}-\lambda_{n}^{+}\right)}\left[\frac{\lambda_{n}^{-}+{\left(\pi n\right)}^{2}\Delta }{\lambda_{n}^{+}}e^{-\lambda_{n}^{+}\tau }-\frac{\lambda_{n}^{+}+{\left(\pi n\right)}^{2}\Delta }{\lambda_{n}^{-}}e^{-\lambda_{n}^{-}\tau }\right]\right\}$$

where

(12) $$t_{i}=\frac{\tau_{i}}{6}\frac{\beta +\alpha }{\beta +\alpha \varepsilon }$$

(13) $$\lambda_{n}^{\pm }\left(=\right)\frac{\left[\left(\varepsilon +1\right){\left(\pi n\right)}^{2}+\left(\beta +\alpha \right)\right]}{2}\pm \sqrt{\frac{{\left[\left(\varepsilon +1\right){\left(\pi n\right)}^{2}+\left(\beta +\alpha \right)\right]}^{2}}{4}-{\left(\pi n\right)}^{2}\left[\varepsilon {\left(\pi n\right)}^{2}+\left(\beta +\alpha \varepsilon \right)\right]}$$

Essential is the fact that at large times t→∞, the dependence of M on *t* is approximated by a straight line

(14) $$M_{\left(t\to \infty \right)}=\frac{mD_{i}+\left(1-m\right)D_{a}}{l}c_{0}\left(t-t_{1}\right)$$

And upon extrapolation *t* → 0, this line, in total accordance with the obtained experimental material, does not go to the origin of coordinates, but crosses the time axis at the values of

(15) $$t_{1}\equiv \Theta =\frac{l^{2}\left(m+\gamma \left(1-m\right)\right)}{6\left(D_{i}m+\gamma \left(1-m\right)D_{a}\right)}$$

and ordinate axis at

(16) $$M_{\left(t\to 0\right)}=\frac{l}{6}\left(m+\gamma \left(1-m\right)\right)c_{0}$$

From Equations (12)–(16) it is clear that the obtained equations are more general than the Dynes–Barrer ratio [9]. They are very satisfactory in describing of the obtained set of experimental data. Thus, it follows from Equation (15) that the length of the non-stationary stage of the process of permeability is a function of the membrane porosity, its sorption capacity by diffusant, the ratio between the diffusion coefficients in the porous space and polymeric material. It is natural that in general case, upon consideration of ${\left(\frac{\partial M}{\partial T}\right)}_{t\to \infty }$ or $\left(\frac{\partial \Theta }{\partial T}\right)\;$it is necessary to expect a quite complex temperature dependence of the mentioned process parameters:

(17) $$\left(\frac{\partial I}{\partial T}\right)=const\left(\frac{\partial D_{i}}{\partial T}\right)+const\left(\frac{\partial D_{a}}{\partial T}\right)+const\left(\frac{\partial \gamma }{\partial T}\right)$$

which can be approximated by $I\approx T^{-n}$ or an exponential function only in the first approximation at certain relations between D_i_, D_a_, and σ.

Thus, the new possibility of calculating diffusion coefficients from kinetic sorption curves of monolithic samples and kinetic permeability curves of porous membranes, supplemented with information on porosity, is of great interest. For this purpose, it is possible to use either the ratio

$$D_{i}m=\frac{l}{c_{0}\theta }{\left(-M\right)}_{t\to 0}-D_{a}\gamma^{©}\left(1-m\right)$$

(18) $$D_{a}=\frac{a_{1}-a_{2}}{\left(\gamma_{1}^{©}-\gamma_{2}^{©}\right)\left(1-m\right)}$$

$$D_{i}m=a_{1}-\lambda_{1}^{©}\left(\frac{a_{1}-a_{2}}{\gamma_{1}^{©}-\lambda_{2}^{©}}\right)$$

or, carrying out the measurements of I at different γ′, i.e., at different humidity differences. In this case:

(19) $$\begin{array}{c} a_{1}=D_{i}m+\sigma_{1}D_{a}\left(1-m\right)\\ a_{2}=D_{i}m+\sigma_{2}D_{a}\left(1-m\right) \end{array}$$

where $\;a_{i}=I_{i}l{\left(c_{0}\right)}_{i}^{-1}$.

Calculations by these equations for all membranes have shown that the transfer of water vapor with a diffusion coefficient of D_i_ = 0.18 − 0.21 at 298 K occurs in their pore space, which is characteristic of the free diffusion of water vapor in air.

## 4. Conclusions

Thus, in the framework of the bidisperse Zolotarev–Dubininin model, an analytical equation, which connects permeability with water vapor diffusion coefficients in the pore space of the membrane, polymer frame material, porosity, and solubility coefficient, is derived. The obtained analytical ratios can be widely used to predict and calculate various situations arising from the operation of real porous polymeric membranes. The use of this equation is proposed to predict the sorption properties of barrier shells and porous materials of complex architectures, for example, in the analysis of mass transfer in disperse mixtures of polymers, block copolymers, gradient barrier layers. It should be noted that the proposed analytical equations allow one to also solve the inverse problem to estimate structural and defective characteristics of a given polymer membrane [14,15].

## Figures and Tables

**Table 1:** Characteristics and kinetic constants of diffusion and permeability of porous polymeric membranes of different hydrophilicity at 298 K.

	Material	PVC	PEU-1	PEU-2	PEU-3	Collagen and Natural Leather
Parameter						
Porosity, %		38	55	58	65	44
Pore diameter, µm		8/12 ***	5/8	7/11	6/10	4/6
Water sorption, g/100 g		0.52	7.5	9.2	16.3	60
D_Θ_, *10^−7^ cm^2^/s		2	0.8	0.6	4	0.1
D_s_, *10^−7^ cm^2^/s		3.1	0.66	0.5	2.4	0.08
D_p_, *10^−2^ cm^2^/s *		~1	~1	~1	~1	~1
E_Θ_, kJ/mol		51/52 **	48/46	54/49	56/54	61/63
E_S_, kJ/mol		50/54	49/48	53/50	52/50	64/62
E_P_, kJ/mol		54/29	51/25	57/18	58/21	63/26

* Calculated by ratio D_P_ = P/σ. σ is obtained from a real isotherm. ** In the numerator, the activation energy at a partial pressure difference of 44–60%, and in the denominator at a partial pressure difference of 44–90%. *** The range of pore size changes.
